# Risk factors for adverse reactions caused by abemaciclib in breast cancer therapy

**DOI:** 10.3389/fonc.2025.1529980

**Published:** 2025-06-20

**Authors:** Xianghua Quan, CaiHong Sun, Bing Han, ChuanZhou Zhang, HuaiQin Cang, Xiaomin Xing, Qie Guo

**Affiliations:** ^1^ Department of Clinical Pharmacy, The Affiliated Hospital of Qingdao University, Qingdao, Shandong, China; ^2^ Department of Clinical Pharmacy. Shaoxing Second Hospital, Shaoxing, China

**Keywords:** abemaciclib, breast cancer, diarrhea, neutropenia, CDK4/6 kinase

## Abstract

**Introduction:**

In recent years, a range of cyclin-dependent kinase 4/6 (CDK4/6) inhibitors have been identified as significantly improving the survival of patients with hormone receptor-positive (HR+)/human epidermal growth factor receptor 2-negative (HER2-) breast cancer (BC). As the use of CDK4/6 inhibitors continues to increase, safety concerns have garnered increasing attention. Herein, this study analyzed adverse reactions in breast cancer patients receiving a CDK4/6 inhibitor abemaciclib, with a focus on identifying risk factors for diarrhea and neutropenia through regression analysis.

**Methods:**

In this study, a total of 216 BC patients receiving abemaciclib were enrolled. Follow-up observations towards the baseline and clinical characteristics in these patients were exhibited. The evaluation of adverse effects (AEs) in these patients was performed based on the clinical practice of abemaciclib whole-course management and the consensus on the management. Subsequently, we focused on the two most common adverse reactions during the use of abemaciclib, namely diarrhea and neutropenia. Furthermore, analysis of factors influencing incidence of diarrhea and neutropenia was executed using the univariate analysis and multivariate logistic regression analysis.

**Results:**

The safety profile of abemaciclib was manageable, and the drug was well tolerated by patients. The incidence of AEs was greater in the gastrointestinal system, blood and lymphatic system, liver system, renal system, muscular and skeletal systems, and skin and subcutaneous tissue systems. Age stratification and gastrointestinal diseases were independent risk factors for grade 2-3 diarrhea. Alternatively, the Eastern Cooperative Oncology Group (ECOG) score was a factor associated with the risk for grade 3-4 neutropenia. Baseline BMI classification, baseline white blood cell (WBC) count and baseline albumin (ALB) stratification were factors associated with protection against grade 3-4 neutropenia.

**Discussion:**

This study retrospectively collected, processed, analyzed, and evaluated the safety profile of abemaciclib. Additionally, potential influencing factors associated with common adverse reactions including diarrhea and neutropenia were explored to provide a foundation for its rational clinical application.

## Introduction

Breast cancer (BC) is the cancer with the highest morbidity and mortality in females and seriously jeopardizes women’s physical and mental health (1). BC is highly heterogeneous, and HR+/HER2- is the most common molecular subtype, accounting for approximately 75% of BC cases (2). Endocrine therapy provides several advantages, including low drug toxicity, relatively affordable costs, and convenient administration. As a well-established treatment modality for BC, endocrine therapy is frequently employed to treat hormone receptor-positive/human epidermal growth factor receptor 2-negative (HR+/HER2-) BC. However, some patients exhibit primary or secondary resistance to endocrine therapy as a result of the activation of the hormone receptor signaling pathway (3). Abemaciclib is currently the sole CDK4/6 inhibitor approved for the treatment of early-stage BC. It inhibits the activity of CDK4/6 kinase, thereby preventing the transition of the cell cycle from the G1 phase to the S phase and suppressing tumor cell proliferation. Additionally, abemaciclib targets the estrogen receptor signaling pathway, creating a synergistic effect when combined with endocrine therapy (4). Abemaciclib has been shown to significantly improve the survival rate of BC patients and has emerged as a novel treatment option for hormone receptor-positive/human epidermal growth factor receptor 2-negative (HR+/HER2-) BC patients. Consequently, the introduction of abemaciclib marks a transformative shift from conventional chemotherapy or endocrine monotherapy to innovative targeted combination therapy, heralding a new era in targeted therapy for HR+/HER2- BC (5).

Blood toxicity and diarrhea are the most frequently reported adverse effects (AEs) associated with CDK4/6 inhibitors. Notably, abemaciclib may induce hepatotoxicity and nephrotoxicity, which can compromise patient tolerance and hinder the attainment of maximal therapeutic efficacy. Furthermore, these toxicities may predispose patients to severe infections, thereby posing significant risks to their health and, in extreme cases, threatening their lives (6, 7). Therefore, it is essential to screen patients for risk factors associated with common abemaciclib-related AEs and implement preventive measures and early interventions to mitigate their impact on patients. In this study, comprehensive patient information, including clinical characteristics, laboratory indicators, and medications, was systematically collected. Patients were closely monitored for adverse reactions during treatment, and multivariate logistic regression analysis was conducted on clinical characteristics and laboratory indicators to identify the factors influencing common abemaciclib-related AEs.

## Materials and methods

### Subject

Initially, 447 breast cancer patients who received abemaciclib treatment at the Affiliated Hospital of Qingdao University came into our view. Following stringent inclusion and exclusion criteria (as detailed below), a total of 216 patients were ultimately included in this study, while the remaining 231 patients were excluded due to data loss, loss to follow-up, concomitant medication use, and other reasons.

### Inclusion and exclusion criteria

The following patients were enrolled: (1) female aged ≥18 years old; (2) breast cancer confirmed by pathology; (3) Estrogen receptor (ER) or Progesterone receptor (PR) positivity confirmed by histology or cytology (defined as 1% cell positivity); (4) The treatment regimen is abemaciclib combined with endocrine therapy; (5) Eastern Cooperative Oncology Group (ECOG) score ≤3; (6) Complete medical records and complete baseline data, with complete blood count, liver and kidney function tests during treatment. The following patients with other primary tumors or incomplete data should be excluded in this study.

### Flow chart of this study

**Figure 1:**
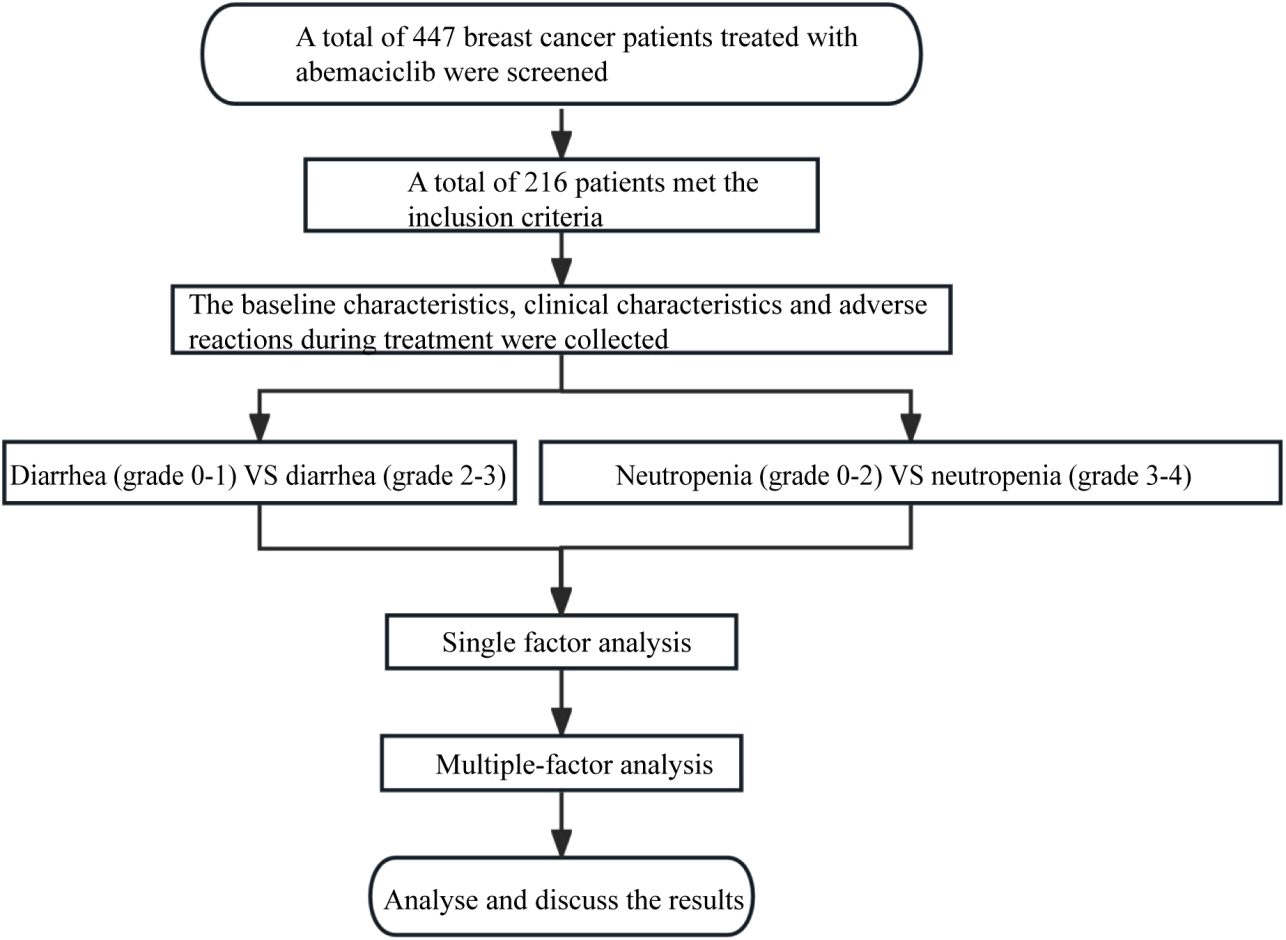
Flow chart of research development.

Flow chart of research development was show in **Figure 1**. This study was approved by the Ethics Committee of Qingdao University (Approve number: QDFY-EC-2021-0156), and written informed consent was obtained from each patient. The study was in compliance with the Declaration of Helsinki.

### Therapeutic regimen

Abemaciclib is initially recommended at a dose of 150 mg orally twice daily, with subsequent dose reductions of 50 mg increments as needed based on patient tolerability. Endocrine therapeutic drugs, including selective estrogen receptor modulators (e.g., tamoxifen and toremifene), aromatase inhibitors (e.g., anastrozole, letrozole, and exemestane), and selective estrogen receptor downregulators (e.g., fulvestrant), were utilized in this study. The drug is administered until disease progression occurs, the patient is unable to tolerate the side effects, discontinues the treatment, or passes away.

### Follow-up observations

The clinical characteristics of the patients were collated and the occurrence of adverse reactions in these patients was documented. In particular, these following observational index were determined:

1. Basic information: name, BMI, ECOG score, past medical history, treatment stage;
2. Past medical history: diabetes mellitus, hypertension, gastrointestinal disease, hyperlipidemia, osteoporosis, hyperuricemia, thyroid disease, etc.
3. Laboratory examination information: baseline ALB, baseline WBC, baseline NEUT, baseline PLT, baseline AMC, baseline ALC, baseline LMR, baseline NLR, and baseline PLR before treatment;
4. Pathological information: ER, PR, HER2 and Ki-67.

### Assessment criteria of AEs

Current research findings indicate that hot flashes, night sweats, and other symptoms associated with menopausal syndrome, along with fatigue and bone and joint pain, are the most prevalent adverse reactions experienced by breast cancer patients undergoing endocrine therapy (8). Thus, the AEs in these patients were considered as in response to abemaciclib are the result of monotherapy although they received a combination treatment of abemaciclib and endocrine therapy agents. According to the clinical practice of abemaciclib whole-course management and the consensus on the management of AEs related to CDK4/6 inhibitors in breast cancer (9), diarrhea and neutropenia occurred with abemaciclib were graded, as shown in **Tables 1** and **2**.

**Table 1:** Classification criteria for diarrhea.

Classification	Clinical Manifestation
Grade 1	Defecation frequency ≤3 times per day compared with baseline; slight increase in stoma output.
Grade 2	Defecation frequency 4-6 times per day compared with baseline; moderate increase in stoma output; limitations in daily activities requiring assistive tools.
Grade 3	Defecation frequency ≥7 times per day compared with baseline; severe increase in stoma output; hospitalization required; limited self-control in daily life.
Grade 4	Life-threatening; urgent intervention necessary.
Grade 5	Death.

**Table 2:** Classification criteria for hematologic toxicity.

Classification	Grade 1	Grade 2	Grade 3	Grade 4
The number of Neutrophils	≥1.5×10^9^/L and <lower limit of normal value	≥1.0×10^9^/L and <1.5×10^9^/L	≥0.5×10^9^/L and<1.0×10^9^/L	<0.5×10^9^/L

For grade ≥ 2 diarrhea, dose reduction or treatment discontinuation should be considered if the condition persists or recurs (10). Diarrhea induced by abemaciclib is generally characterized as mild to moderate and resolves promptly in most cases, with many patients demonstrating tolerability. However, in some instances, it may lead to decreased compliance, treatment delays, or missed doses, thereby potentially compromising the drug’s overall efficacy (11). Therefore, it is essential to identify patients who are at risk of experiencing grade ≥ 2 diarrhea as early as possible in real-world settings.

Neutropenia is a significant limiting toxicity associated with CDK4/6 inhibitors and ranks among the most prevalent side effects (12). Grade ≥ 3 neutropenia necessitates either drug suspension or dose reduction during treatment, and it may be complicated by sepsis (13), which can adversely impact both drug efficacy and patients’ quality of life. Consequently, this study stratified participants based on the occurrence of grade ≥ 3 neutropenia to investigate its influencing factors.

### Statistical analysis

Statistical analysis was performed on the relevant data using SPSS 26.0 software. Quantitative data that followed a normal distribution were presented as X ± S (mean ± standard deviation). Non-normally distributed data were analyzed using the non-parametric rank sum test and expressed as interquartile range (IQR). Qualitative data were evaluated using the χ2 test and reported as counts and percentages. Univariate analysis was performed on the demographic and clinical information of the included patients, and variables with P<0.05 were further incorporated into multivariate Logistic regression analysis.

## Results

### Baseline conditions and examination indicators

A total of 216 breast cancer (BC) patients treated with abemaciclib were included in this study. Specifically, the cohort consisted of 201 patients younger than 70 years old and 15 patients aged 70 years or older; 108 patients with a body mass index (BMI) below 24 and 108 patients with a BMI of 24 or higher; 6 patients with an Eastern Cooperative Oncology Group (ECOG) performance status score of 0, 187 patients with an ECOG score of 1, and 2 patients with an ECOG score of 2; 138 patients classified as luminal A subtype and 78 patients classified as luminal B subtype based on molecular classification; 16 patients with gastrointestinal diseases and 200 patients without gastrointestinal diseases; 50 patients who received postoperative adjuvant therapy, 166 patients who received treatment for advanced-stage BC, and 27 patients with baseline albumin (ALB) levels below 4.0 g/dL, while 189 patients had baseline ALB levels of 4.0 g/dL or higher. The baseline median white blood cell (WBC) count was 4.57, the baseline median neutrophil (NEUT) count was 2.69, the baseline median platelet (PLT) count was 207.5, the baseline median absolute monocyte count (AMC) was 0.34, the baseline median absolute lymphocyte count (ALC) was 1.38, the baseline median lymphocyte-to-monocyte ratio (LMR) was 4.13, the baseline median neutrophil-to-lymphocyte ratio (NLR) was 1.92, and the baseline median platelet‐to‐lymphocyte ratio (PLR) was 152.16. More details were shown in **Table 3**.

**Table 3:** Table of basic information of patients.

Clinical features	N (%)/X ± S/M(Q25-Q75)
Age stratification		
<70	201
≥70	15
BMI (Kg/m^2^)		
<24	108
≥24	108
ECOG score		
0	6
1	187
2	23
Molecular subtyping		
LuminalA	138
LuminalB	78
Presence of gastrointestinal disease		
Yes	16
No	200
Phase of treatment		
Assist	50
Later period	166
Stratification of baseline ALB (g/L)		
<4.0	27
≥4.0	189
Baseline WBC (×10^9^/L)	4.57 (3.79-5.77)
Baseline NEUT (×10^9^/L)	2.69 (2.00-3.53)
Baseline PLT (×10^9^/L)	207.5 (160.25-252)
Baseline AMC (×10^9^/L)	0.34 (0.25-0.46)
Baseline ALC (×10^9^/L)	1.38 (1.05-1.82)
Baseline LMR	4.13 (2.84-5.79)
Baseline NLR	1.92 (1.32-2.79)
Baseline PLR	152.16 (104.13-208.63)

Lymphocyte/monocyte ratio (LMR) = ALC/AMC; Neutrophil/lymphocyte ratio (NLR) =NEUT/ALC; Platelet/lymphocyte ratio (PLR) =PLT/ALC.

### Category and number of adverse events

The AEs of 216 patients who received abemaciclib treatment were summarized and shown in the **Table 4**. The gastrointestinal system, hematological system, lymphatic system, liver function, kidney function, muscular and skeletal systems, metabolic and nutritional systems, as well as the nervous system were affected. Overall, the safety profile of abemaciclib was manageable, and the drug was well tolerated by patients. The incidence of AEs was greater in the gastrointestinal system, blood and lymphatic system, liver system, renal system, muscular and skeletal systems, and skin and subcutaneous tissue systems. There were 177 patients with diarrhea (82.0%), 67 patients with nausea (31.0%), 42 patients with vomiting (19.4%), 159 patients with neutropenia (73.6%), 159 patients with leukopenia (73.6%), 143 patients with anemia (66.2%), 77 patients with thrombocytopenia (35.6%), 64 patients with elevated transaminases (29.6%), 22 patients with increased total bilirubin levels (10.2%), 93 patients with increased uric acid levels (43.1%), 113 patients with fatigue (52.3%)%, 43 patients with pruritus (19.9%), and 42 patients with rash (19.4%).

**Table 4:** Classification and grading of adverse effects.

Types of adverse reactions	Total number of cases (%)	Number of cases of grade I-II adverse reactions (%)	Number of cases of grade III-IV adverse reactions (%)
Gastrointestinal diseases			
Diarrhea	177 (82.0%)	157 (72.7%)	20(9.3%)
Nausea	67 (31.0%)	67 (31.0%)	0
Emesis	42 (19.4%)	42 (19.4%)	0
Blood and lymphatic system			
Neutropenia	159 (73.6%)	109 (50.5%)	50 (23.1%)
Leukopenia	159 (73.6%)	125 (57.9%)	34 (15.7%)
Anemia	143 (66.2%)	127 (58.8%)	16 (7.4%)
Thrombocytopenia	77 (35.6%)	68 (31.5%)	9 (4.2%)
Lymphopenia	103 (47.7%)	103 (47.7%)	0
Liver function			
Elevation of transaminase	64 (29.6%)	58 (26.9%)	6 (2.8%)
Elevation of the total bilirubin	22 (10.2%)	17 (7.9%)	5 (2.3%)
Renal function			
Elevation of uric acid	93 (43.1%)	93 (43.1%)	0
Elevation of creatinine	16 (7.4%)	15 (7.0%)	1 (0.5%)
Systemic system			
Lack of strength	113 (52.3%)	113 (52.3%)	0
Metabolic and nutritional diseases			
Loss of appetite	30 (13.9%)	30 (13.9%)	0
Various diseases of the nervous system			
Dizzy	31 (14.4%)	31 (14.4%)	0
Agrypnia	36 (16.7%)	36 (16.7%)	0
Eye organ diseases			
Increased tear	7 (3.2%)	7 (3.2%)	0
Vascular and lymphatic diseases			
Venous thromboembolism	9 (4.2%)	9 (4.2%)	0
Diseases of the respiratory system	24 (11.1%)	24 (11.1%)	0
Diseases of the skin and subcutaneous tissue			
Alopecia	10 (4.6%)	10 (4.6%)	0
Pruritus	43 (19.9%)	43 (19.9%)	0
Erythra	42 (19.4%)	42 (19.4%)	0
Xerodermia	11 (5.1%)	11 (5.1%)	0

### Univariate analysis for risk factors between diarrhea (grades 0-1) and diarrhea (grades 2-3)

In this study, there were 147 cases of grade 0-1 diarrhea and 69 cases of grade 2-3 diarrhea. Univariate analysis indicated that the differences in age (P = 0.007) and gastrointestinal diseases (P=0.001) were statistically significant. However, no significant differences were observed in BMI, ECOG score, molecular classification, treatment stage, baseline ALB stratification, baseline WBC count, baseline NEUT count, baseline PLT count, baseline AMC, baseline ALC, baseline LMR, baseline NLR, or baseline PLR (**Table 5**).

**Table 5:** Univariate analysis of diarrhea (grade 0-1) group and diarrhea (grade 2-3).

Influence factor	Diarrhea (grade 0-1)	Diarrhea (grade 2-3)	*P*
Number of cases	147 (68.1%)	69 (31.9%)	
Age stratification			0.007*
<70	142 (96.6)	59 (93.1)	
≧70	5 (3.4)	10 (6.9)	
BMI(Kg/m^2^)			0.307
<24	77 (52.4)	31 (44.9)	
≧24	70 (47.6)	38 (55.1)	
ECOG score			0.062
0	6 (4.1)	0 (0)	
1	129 (87.8)	58 (84.1)	
2	12 (8.2)	11 (15.9)	
Molecular subtyping			0.349
Luminal A	97 (66.0)	41 (59.4)	
Luminal B	50 (34.0)	28 (40.6)	
Gastrointestinal disease			0.001*
Yes	5 (3.4)	11 (15.9)	
No	142 (96.6)	58 (84.1)	
Phase of treatment			0.992
Assistance	34 (23.1)	16 (23.2)	
Later period	113 (76.9)	53 (76.8)	
Baseline ALB (g/L)			0.295
<4.0	16 (10.9)	11 (15.9)	
≥4.0	131 (89.1)	58 (84.1)	
Baseline WBC (×10^9^/L)	4.58 (3.9-5.96)	4.55 (3.3-5.64)	0.71
Baseline NEUT (×10^9^/L)	2.62 (1.95-3.51)	2.77 (2.08-3.54)	0.756
Baseline PLT (×10^9^/L)	210 (165-255)	203 (155-245)	0.132
Baseline AMC (×10^9^/L)	0.35 (0.26-0.47)	0.34 (0.25-0.45)	0.248
Baseline ALC (×10^9^/L)	1.37 (1.04-1.84)	1.39 (1.08-1.78)	0.82
Baseline LMR	4.09 (2.71-5.79)	4.43 (3.16-5.82)	0.279
Baseline NLR	1.87 (1.29-3.04)	1.99 (1.48-2.56)	0.657
Baseline PLR	158.96 (105.86-221.51)	146.6 (99.52-189.26)	0.133

A P-value of less than 0.05 (P*<0.05) is considered statistically significant.

### Multivariate logistic regression analysis for risk factors between diarrhea (grades 0-1) and diarrhea (grades 2-3)

As previously outlined, this study performed a univariate analysis focusing on age stratification, BMI, ECOG score, molecular typing, gastrointestinal diseases, treatment stage, baseline ALB stratification, baseline WBC count, baseline NEUT count, baseline PLT count, baseline AMC count, baseline ALC count, baseline LMR ratio, baseline NLR ratio, and baseline PLR ratio. The results demonstrated that the p-values for both age stratification and gastrointestinal diseases were less than 0.05. Subsequently, a multivariate logistic regression analysis was conducted to further investigate these significant factors. In this analysis, the occurrence of diarrhea (grades 2-3) was designated as the dependent variable, while age stratification and gastrointestinal diseases were treated as independent variables. The results revealed that age stratification (*P*=0.021) and gastrointestinal diseases (*P*=0.009) were independent risk factors for grade 2-3 diarrhea. The incidence of 2-3 diarrhea in patients with gastrointestinal diseases was 4.499 times greater than that in patients without gastrointestinal diseases (**Table 6**).

**Table 6:** Diarrhea (grade 0-1) group and diarrhea (grade 2-3) multivariate analysis.

Influence factor	B	S.E	Wald	Sig	Exp (B)	The 95% confidence interval for EXP (B)	
						Lower limit	Upper limit
Age stratification	1.361	0.59	5.323	0.021	3.901	1.227	12.4
Gastrointestinal disease	1.504	0.577	6.786	0.009	4.499	1.451	13.946

### Univariate analysis for risk factors between neutropenia (grade 0-2) and neutropenia (grade 3-4)

In this study, 166 patients exhibited grade 0-2 neutropenia, while 50 patients experienced grade 3-4 neutropenia. Univariate analysis identified BMI classification (P=0.010), ECOG score (P=0.001), and baseline albumin (ALB) stratification (P = 0.001) as potential factors associated with the development of severe neutropenia (grade 3-4). Specifically, the baseline WBC count was 4.74 in the grade 0-2 neutropenia group and 4.32 in the grade 3-4 neutropenia group (P=0.004). Similarly, the baseline NEUT count was 2.77 in the grade 0-2 neutropenia group and 2.33 in the grade 3-4 neutropenia group (P=0.002). These differences were statistically significant. However, no significant differences were observed in age stratification, BMI, ECOG score, molecular classification, gastrointestinal diseases, treatment stage, baseline PLT count, baseline AMC, baseline ALC, baseline LMR, baseline NLR, or baseline PLR (**Table 7**).

**Table 7:** Univariate analysis of the neutropenia (grade 0-2) group versus neutropenia (grade 3-4).

Influence factor	Neutropenia (grade 0-2)	Neutropenia (grade 3-4)	*P*
Number of cases	166 (76.9%)	50(23.1%)	
Age stratification			0.738
<70	155 (93.4)	46 (92.0)	
≥70	11(6.6)	4(8.0)	
BMI(Kg/m^2^)			0.010*
<24	75 (45.2)	33 (66.0)	
≥24	91 (54.8)	17 (34.0)	
ECOG score			0.001*
0	6 (3.6)	0	
1	149 (89.8)	38 (76.0)	
2	11 (6.6)	12 (24.0)	
Molecular subtyping			0.323
LuminalA	109 (65.7)	29 (58.0)	
LuminalB	57 (34.3)	21 (42.0)	
Presence of gastrointestinal disease			0.900
Yes	13 (7.8)	3 (6.0)	
No	153 (92.2)	47 (94.0)	
Phase of treatment			0.871
Assistance	38 (22.9)	12 (24.0)	
Later period	128 (77.1)	38 (76.0)	
Stratification of baseline ALB (g/L)			0.001*
<4.0	14 (8.4)	13 (26.0)	
≥4.0	152 (91.6)	37 (74.0)	
Baseline WBC(×10^9^/L)	4.74 (3.95-6.11)	4.32 (3.40-4.83)	0.004*
Baseline NEUT(×10^9^/L)	2.77 (2.16-3.86)	2.33 (1.71-3.02)	0.002*
Baseline PLT(×10^9^/L)	210 (164.75-253.25)	203.20 (147.25-239.25)	0.131
Baseline AMC(×10^9^/L)	0.34 (0.26-0.48)	0.34 (0.24-0.41)	0.169
Baseline ALC(×10^9^/L)	1.41 (1.04-1.85)	1.28 (1.07-1.63)	0.420
Baseline LMR	4.10 (2.70-5.81)	4.38 (2.97-5.79)	0.589
Baseline NLR	1.99 (1.35-3.13)	1.77 (1.18-2.32)	0.053
Baseline PLR	153.53 (105.41-212.61)	150.91 (101.14-201.22)	0.479

A P-value of less than 0.05 (P*<0.05) is considered statistically significant.

### Multivariate logistic regression analysis for risk factors between neutropenia (grade 0-2) and neutropenia (grade 3-4)

As previously outlined, this study conducted a univariate analysis on multiple factors, including age stratification, BMI classification, ECOG score, molecular typing, history of gastrointestinal diseases, treatment stage, baseline ALB stratification, baseline WBC count, baseline NEUT count, baseline PLT count, baseline AMC count, baseline ALC count, baseline LMR ratio, and baseline NLR ratio. The results demonstrated that the P-values for BMI classification, ECOG score, baseline ALB stratification, as well as the counts of baseline WBC and NEUT were all less than 0.05. Factors with P-values less than 0.05 were subsequently included in multivariate logistic regression analysis. In this analysis, where the occurrence of grade 3-4 neutropenia served as the dependent variable, collinearity among influencing factors was excluded based on prior research findings (14), resulting in the removal of similar influencing variables. Ultimately, BMI classification, ECOG score, baseline ALB stratification, and baseline WBC count were selected as independent variables for further investigation. The multivariate logistic regression revealed that the ECOG score (*P* = 0.008) was a factor associated with the risk for grade 3-4 neutropenia, and baseline BMI classification (*P* = 0.035), baseline WBC count (*P* = 0.007), and baseline ALB stratification (*P* = 0.031) were factors associated with protection against grade 3-4 neutropenia (**Table 8**).

**Table 8:** Multivariate analysis of the neutropenia (grade 0-2) group versus neutropenia (grade 3-4).

Influence factor	B	S.E	Wald	Sig	Exp(B)	The 95% confidence interval for EXP (B)	
						Lower limit	Upper limit
BMI Classification	-0.757	0.359	4.451	0.035	0.469	0.232	0.948
ECOG score	1.265	0.476	7.073	0.008	3.545	1.395	9.007
Baseline WBC	-0.341	0.127	7.279	0.007	0.711	0.555	0.911
Stratification of baseline ALB	-0.994	0.461	4.661	0.031	0.37	0.15	0.912

## Discussion

During treatment with abemaciclib, gastrointestinal toxicity particularly diarrhea is the most common AE in patients with BC. In the phase II MONARCH1 trial (15), diarrhea occurred in 90.2% of patients, with 19.7% experiencing grade ≥3 severity and 20.5% requiring dose reductions. In phase III MONARCH2 (16) and MONARCH3 (17) trials, the incidences of diarrhea were 85.4% and 81.1%, respectively, while grade ≥3 events occurred in 13.4% and 9.3% of cases. The MONARCH2 and MONARCH3 studies (10) further indicated that diarrhea associated with abemaciclib combined with endocrine therapy peaked during in the first month of treatment and then declined over time. The median duration of the initial episode of diarrhea was 6-8 days;for grade 2, 9–12 days; and for grade 3, 6–8 days. In the MONARCHE study on adjuvant therapy (18), the incidence of diarrhea caused by abemaciclib combined with endocrine therapy was 83.5%, with grade ≥3 events occurring in 7.8% of patients. The median time to onset was 8 days, and the median duration was ≤7 days. In the subsequent multinational phase III MONARCHplus (19) study, 78-80% of patients experienced diarrhea, which was generally mild; only 2-4% developed grade ≥3 events. However, Hamilton E et al. (20) showed that for the abemaciclib treatment, the real-world incidence of diarrhea was reduced to 43%-67%, and fewer than 10% of the patients had grade ≥3 diarrhea. Similarly, Cuyun Carter et al. (21) evaluated the efficacy of abemaciclib in HR+/HER2- advanced BC patients within the first year after the Food and Drug Administration (FDA) approval and found a 67% incidence of diarrhea. These results may be related to the preventive use of antidiarrheal drugs. In this study, the incidence of diarrhea in patients was 67%, and the incidence of grade ≥3 diarrhea was 9.3%; these values are consistent with the findings of these clinical trials.

Compared with other CDK4/6 inhibitors, the gastrointestinal toxicity associated with abemaciclib is more pronounced and involves multiple contributing factor. In addition to the inhibition of CDK4 and CDK6, abemaciclib also targets CDK9, a key regulator of intestinal cell proliferation, thereby increasing the incidence of diarrhea. In a preclinical model (22), morphological changes in the gastrointestinal tract, such as severe absence of microvilli, vacuolar degeneration, reduction of goblet cells, shortening of villi, proliferation of crypt cells, enterocyte degeneration, and mucosal inflammation, were observed in abemaciclib-treated rats. Moreover, abemaciclib has been shown to activate the Wnt/β-catenin pathway and upregulate the expression of solute carrier family genes such as SLC28a1,SLC37a2, and SLC5a12, ultimately promoting cell proliferation (23). Abemaciclib also inhibits GSk3β and CAMKII to increase intestinal peristalsis, thus causing diarrhea (24).

Therefore, in this study, age, BMI, comorbid gastrointestinal diseases, and inflammatory factors were included in the investigation of diarrhea-associated risk factors. The analyses revealed that the occurrence of grade ≥2 diarrhea was significantly correlated with age ≥ 70 years (*P* = 0.007) and comorbid gastrointestinal disease (*P* = 0.001). If patients are ≥70 years old or have gastrointestinal diseases, the chance of developing grade ≥2 diarrhea greatly increases. To date, few studies have investigated the risk factors associated with abemaciclib treatment. This study revealed that the risk factors for diarrhea were age ≥ 70 years and comorbid gastrointestinal diseases. Modi ND et al. (25) used Cox proportional hazards analysis to study the correlations between pretreatment clinicopathological data and the development of grade ≥3 diarrhea after treatment in the MONARCH1, MONARCH2, and MONARCH3 clinical trials, and the analysis revealed that age ≥70 years was a risk factor for the development of grade ≥3 diarrhea induced by abemaciclib, which is related to decreased gastrointestinal function and disorders caused by ageing (26). Furthermore, polypharmacy and pharmacokinetic/pharmacodynamic changes in the elderly subgroup may lead to an increased risk of abemaciclib-induced grade ≥3 diarrhea (27, 28). In addition, abemaciclib may cause mucosal inflammation, thus inducing diarrhea (22). Findings of other studies (29) have indicated that CDK4/6 inhibitors may attenuate CDK6-dependent inflammatory gene expression. The LMR, NLR and PLR are markers of body inflammation, which can lead to tissue and cell damage and increased vascular permeability, and tumor-associated inflammation, which promotes tumorigenesis, angiogenesis and tumor progression (30). Therefore, inflammatory factors, such as the baseline LMR, NLR, and PLR, were included in this study, but no correlation was found between these factors and grade ≥2 diarrhea, since the diarrhea caused by abemaciclib is considered to be related mainly to the stimulation of a secondary target (31). In addition, Franzoi MA et al. (32) examined the impact BMI on the incidence of diarrhea in the MONARCH2 and MONARCH3 trials and found no statistically significant association consistent with the results of our study. Compared with CDK6, abemaciclib exhibits greater selectivity for CDK4 inhibition (33, 34). CDK6 regulates the expression of cytokines in hematopoiesis (35) and inhibits the expansion of myeloid progenitor cells, thus playing an important role in myeloid differentiation (36). Inhibiting CDK6 can disrupt hematopoietic stem cell differentiation, leading to myelosuppression, which may necessitate dose reductions or even discontinuation of abemaciclib treatment.

In the phase III MONARCH2 trial (16), the incidence of neutropenia was 46.0%; the incidence of grade ≥3 neutropenia was 26.5%; and the incidences of neutropenia in cycles 1 and 2 were 11.6% and 15.8%, respectively. In the phase III MONARCH3 trial (17), the incidence of neutropenia was 43.7%; the incidence of grade ≥3 neutropenia was 21.1%; the incidences of neutropenia in cycles 1 and 2 were 6.4% and 11.0%, respectively; and the incidences of neutropenia were ≤10% in all subsequent cycles. Due to neutropenia, 10%-13% of patients required dose reductions, 16%-17% missed doses, and 1%-3% discontinued treatment. A pooled study of the MONARCH2 and MONARCH3 trials (37) revealed that the median duration of grade ≥3 neutropenia was 29-33 days, the median time to remission was 11-15 days, and the incidence of grade ≥3 neutropenia was approximately 25%. In addition, for patients with grade ≥3 neutropenia, comorbid infection often occurred within 1 week, with an incidence ranging from 1.5% to 4.0%. The incidence of febrile neutropenia was 1%. A retrospective study by Takada S et al. (38) involving 365 Japanese patients treated with abemaciclib found a 75% incidence of neutropenia. Consistent with these findings, our study observed neutropenia in 73.6% of the 216 patients treated with abemaciclib higher than previously reported in the clinical trials. Similar findings are observed for other CDK4/6 inhibitors. For example, in a phase I study of palbociclib combined with letrozole in Japanese patients, 83% of patients developed neutropenia (39), and similar results have been reported for ribociclib (40). Roncato R (41) showed that the reported incidence of neutropenia in Asian patients was greater than that in non-Asian patients, and this finding may be related to body size, dietary habits such as soybean intake, the expression of proinflammatory genes in tumors, and drug-metabolizing enzymes. In this study, grade 1-2 neutropenia was dominant, and grade ≥3 neutropenia accounted for 23.1% of neutropenia cases, which was consistent with the clinical trial data.

Our analysis revealed that grade ≥3 neutropenia was significantly associated with the ECOG score (*P* = 0.008), baseline serum ALB level (*P* = 0.031), baseline WBC count (*P* = 0.007), and BMI classification (*P* = 0.035). First, the ECOG score can reflect a patient’s physical status and tolerance to treatment and is also closely related to AEs in patients after treatment. A pooled clinical study of abemaciclib similarly reported that patients with higher ECOG scores were more likely to develop grade ≥3 neutropenia (42). The results of this study were consistent with those of previous studies, i.e., the higher the ECOG score, the greater the likelihood for the patient having grade ≥3 neutropenia. Other studies have reported that the plasma protein binding rate of abemaciclib is 95% (43), and low levels of ALB lead to a large increase in free drug, resulting in increased drug toxicity (44). Therefore, baseline ALB level was included in this study to explore the relationship with neutropenia. Compared with patients with a baseline ALB level <4.0 g/dL, patients with a baseline ALB level >4.0 g/dL had a lower incidence of neutropenia. Nakatsukasa H (45) reported that among 33 patients with advanced BC treated with abemaciclib, 27.3% developed SAEs, 12.1% of which were hematologic. Notably, no SAEs occurred in patients with baseline ALB level >4.0 g/dL group. Therefore, a baseline ALB level >4.0 g/dL is expected to be a useful AE marker for the selection of patients for abemaciclib treatment. Abemaciclib should be selected for patients with poor physical strength and liver and kidney diseases only after a comprehensive evaluation is performed, and these patients should be closely monitored during treatment.

Emerging studies demonstrated that the outcomes of BC patients in the high NLR and high PLR groups are worse than those of patients in the low NLR and PLR group. A significantly higher incidence of bone marrow toxicity in the high NLR group than in the low NLR group has been revealed; furthermore, this AR occurs earlier during chemotherapy for lung cancer in the high NLR group than in the low NLR group. Moreover, agranulocytosis and thrombocytopenia after chemotherapy for lung cancer are more severe in the high PLR group than in the low PLR group, and agranulocytosis, decreased hemoglobin, and thrombocytopenia are more likely to occur in the low LMR group. In addition, neutrophils constitute the highest percentage of circulating leukocytes and have a key antibacterial function (33). Therefore, baseline LMR, NLR, and PLR were included in this study, but the results were not statistically significant. It is possible that CDK4/6 inhibitors and chemotherapeutic drugs cause different types of blood toxicity, i.e., CDK4/6 inhibitors inhibit only the cell cycle and do not cause inflammation.

Baseline blood parameters have been identified as important risk factors for the development of neutropenia following treatment with various chemotherapeutic drugs (46, 47). In a pooled clinical study of abemaciclib, Modi ND (25) divided the prebaseline WBC count into four levels: <4.0×109/L, 4.0-4.99×109/L, 5.0-6.5×109/L, and ≥6.5×109/L, and the results revealed that the lower the WBC count in patients before treatment, the greater the incidence of neutropenia. Nakatsukasa H (45) also revealed that the incidence of SAEs in the abemaciclib group with a WBC count >5700/µL was significantly lower than that in the group with a WBC count ≤5700/µL. Iwata H et al. (14) in their analysis of PALOMA-2 and PALOMA-3 clinical trials of palbociclib in Asian populations, also found that patients with lower baseline neutrophil (NEUT) or WBC counts were more likely to develop grade ≥3 neutropenia early during treatment, identifying a NEUT count <3680/mm³ as a predictive risk factor. Baseline blood parameters were also included in this study, and the results showed that the incidence of neutropenia in patients with the low baseline WBC count was high.

It have been shown that anticancer drugs can be dissolved in adipose tissue, thus delaying excretion (48, 49). Other studies also demonstrated that the effect of anticancer drugs is weakened in obese patients (50–52). Therefore, patients BMI was included in this study, and its relationship with grade ≥3 neutropenia was evaluated. Compared with lean and normal body weight patients, overweight and obese patients had a lower incidence of grade ≥3 neutropenia, and the differences were statistically significant. For the MONARCH2 (16) and MONARCH3 (17) clinical trials, Franzoi MA et al. (32) explored the effects of BMI on treatment efficacy and ARs, and their exploration revealed that, compared with patients who were underweight and/or normal weight, overweight and/or obese patients had a statistically significantly lower incidence of any grade of neutropenia and a significantly lower incidence of grade ≥3 neutropenia (14, 50). One possible explanation is that elevated NEUT counts may be serve as inflammatory biomarker in overweight or obese individuals, thereby reflecting a different immunologic baseline (53–55).

Currently, more discussion around potential interventions or clinical actions for high-risk patients has been documented. Diarrhea associated with abemaciclib typically occurs during the early phase of treatment. As the duration of therapy increases, patients generally develop better tolerance, leading to a significant reduction in both the severity and incidence of diarrhea. Therefore, it is advisable to instruct patients to closely monitor and document changes in bowel movement frequency and stool characteristics during the initial weeks of treatment. At present, primary prevention of diarrhea is not recommended (9). For patients at higher risk of such as those with inflammatory bowel disease or irritable bowel syndrome, the dose-escalation strategy of tyrosine kinase inhibitors in the CONTROL study can be referred to, which can effectively reduce the frequency and severity of diarrhea (56). Emerging evidence suggests that biologic agents may increase fecal IgA levels and support the stable longitudinal development of gut microbiota, thereby safeguarding the mucosal surface against pathogenic infections and mitigating inflammatory responses. Mileti E et al. (57) discovered that combining probiotics with loperamide can effectively relieve abemaciclib-induced diarrhea. Furthermore, Masuda H et al. (58) demonstrated that Bifidobacterium, regardless of whether it is combined with trimebutine maleate, can reduce both the duration of diarrhea caused by abemaciclib and the incidence of grade 3 or higher diarrhea, ultimately decreasing the likelihood of drug dose reduction or interruption.

Neutropenia induced by abemaciclib can generally be managed through dose interruptions and appropriate dose adjustment, while endocrine therapy may continue uninterrupted. From a nutritional standpoint, ensuring adequate intake of protein-rich foods such as milk, meat, and eggs is recommended to support neutrophil recovery. To prevent patients from developing febrile neutropenia, it is advisable for all patients to receive pneumococcal vaccination before initiating treatment, along with annual influenza vaccination (59). According to the guidelines of the American Society of Clinical Oncology, all cancer patients undergoing conventional chemotherapy or targeted therapy should be screened for HBV. For individuals infected with HBV, different treatment strategies should be adopted based on their serological characteristics, including measures for treating or preventing viral reactivation (60).

## Conclusion

The incidence of diarrhea and neutropenia associated with abemaciclib is relatively high. Factors such as patient age, comorbidities, ECOG performance status, and baseline indicators may correlate with the occurrence of these two common adverse reactions. This study aims to establish a model based on the aforementioned adverse reaction-related factors, thereby enabling more personalized medication monitoring for patients. Consequently, this approach is expected to enhance patients’ quality of life and potentially extend their survival duration.

## Data Availability

The raw data supporting the conclusions of this article will be made available by the authors, without undue reservation.
